# HOSPICE SERVICES IN THE HOME SETTING AND CHARACTERISTICS OF MEDICARE DECEDENTS

**DOI:** 10.1093/geroni/igz038.1850

**Published:** 2019-11-08

**Authors:** Pauline Karikari-Martin, Lirong Zhao, Lynn Miescier

**Affiliations:** 1 U.S. Public Health Service Commissioned Corps, Rockville, Maryland, United States; 2 Centers for Medicare & Medicaid Services, Baltimore, Maryland, United States

## Abstract

There is little empirical work documenting the characteristics of Medicare beneficiaries receiving hospice services in the home setting using the Medicare place of service codes. The objective of this study is to examine differences in Medicare decedents who received hospice services in traditional and non-traditional homes (assisted living, and nursing home settings) defined by these codes. We conducted a secondary analysis of 675,782 Medicare decedents who received hospice services in 2015. Chi-squared and ANOVA tests were used to describe the socio-demographics, health conditions, utilization, and hospice payments of the decedents. Most of the decedents received hospice care in a traditional home (64.9%), but beneficiaries, aged 85 years and over, received hospice services in assisted living (72.1%) or nursing homes (59.8%). Overall, decedents who received Medicare hospice benefits in assisted living had the highest number of hospice days (mean=149.7 lifetime days; median = 30; standard deviation (S.D.) =245), and decedents in traditional homes had the fewest number of hospice days (mean=86.7 lifetime days; median = 24; S.D. =179). Among Medicare–Medicaid (duals) decedents who used hospice care, 49% received hospice care in nursing homes, and infrequently, 7% at assisted living. Medicare hospice payments were highest ($20,439 per beneficiary) for decedents in assisted living, but least for those in traditional homes ($11,830). Hospice services offered to Medicare beneficiaries who are 85 years and older, duals, or have a diagnosis of dementia may require more oversight and coordination of resources to ensure that they receive appropriate hospice services in non-traditional homes.

